# Measurement and Analysis of Shock Wave Pressure in Moving Charge and Stationary Charge Explosions

**DOI:** 10.3390/s22176582

**Published:** 2022-08-31

**Authors:** Xuejiao Ma, Deren Kong, Yucheng Shi

**Affiliations:** School of Mechanical Engineering, Nanjing University of Science and Technology, Nanjing 210094, China

**Keywords:** shock wave, moving charge explosion, pressure measurement, overpressure distribution

## Abstract

Shock wave pressure is one of the most important parameters in an explosion. However, there have been few experimental and analytical investigations of moving charge explosions. In this article, we present an experimental method to measure the shock wave pressure from a moving charge explosion. Tests of stationary charges and moving charges with speeds of 580 m/s, 703 m/s and 717 m/s were carried out. The shock wave pressure curves and parameters at different measurement points were obtained and analyzed. The theoretical calculation of the shock wave overpressure was studied and compared with the experimental result. The differences between the stationary charge and moving charge explosions were investigated. The results showed that the shock wave pressure distribution of a moving charge had strong directionality. The shock wave pressure parameters (including overpressure, arrival time, duration and impulse) were influenced by the charge’s moving velocity, direction angle and distance from the blast point. The shock wave overpressure value was greater than that of a stationary charge explosion at angles between 0° and 90°. The correlation model based on the velocity vector superposition method could describe the relationship of overpressure between the stationary charge and moving charge explosions.

## 1. Introduction

The shock wave pressure induced by an explosion is one of the main damage parameters; it is usually applied to evaluate the damage range based on the blast overpressure criterion. At present, most of the research on explosion shock waves is based on stationary charge explosions. However, in actual combat, a charge, such as a missile, artillery shell, rocket and other weapons, usually explode during movement. In a stationary charge explosion, the test environment, conditions and spatial relationships are generally consistent and relatively ideal, and the damage parameters present good symmetry. However, the damage power of a moving charge explosion is influenced by the explosion point location, the charge’s moving velocity and orientation, and other factors. A stationary charge explosion experimental study cannot fully reflect the destructive capacity of actual explosion conditions, which is a long-standing problem that has not been addressed. With the development of high-speed weapons, the measurement and analysis of shock waves from moving charge explosions are becoming increasingly important.

A series of studies were conducted to reveal the free-field pressures generated by static explosions [1,2,3,4,5,6]. Many empirical formulae and curves were proposed to predict the overpressure attenuation as a function of the scaled distance and pressure time history of a shock wave. Baker, Brode and Henrych et al. proposed many classical formulae for bare spherical TNT charge explosions in infinite air based on the Sachs scaling law [7,8,9,10]. The measurement of the shock wave pressure of an explosion by using pressure sensors was studied by numerous scholars [11,12,13,14,15]. All the above investigations were aimed at studying the blast effect of stationary charge explosions, but few studies have been done on moving charge explosions.

In the 1950s, Thornhill and Hetherington [16] theoretically analyzed the influence of charge motion on an explosion shock wave. They predicted that close to the surface of a moving charge, the shock velocity, and thus, an inference of the peak pressure could be approximated using vector addition of the shock velocity for the stationary charge and the terminal velocity of the moving charge, but the evaluation of these theories required quantitative data. Subsequently, a series of experiments were carried out at the Ballistic Research Laboratories [17,18,19]. The BRL study focused on the shock wave overpressure of a spherical charge under ideal circumstances, ignoring other parameters, such as the arrival time and impulse.

In recent years, scholars began to carry out numerical simulation studies about moving charge explosions. G. Y. Zhang [20] put forward a method that combined theory with a computer simulation test to study the shock wave characteristics caused by the explosion of a moving charge at a certain height from the ground. They analyzed the distribution characteristics and overpressure time curves of the shock wave. However, this study only considered the shock wave overpressure peak from the charge in the forward and reverse directions. Shang-qing Li [21] studied the shock wave characteristics of an underwater moving explosive. They calculated the underwater peak overpressure in the direction of the explosive motion based on the Cole empirical formula by converting the kinetic energy of the explosive into an equivalent static dose. Yuan Nie [22] numerically investigated the dynamic blast overpressure field using SPEED software. They analyzed the contours of overpressure and peak pressure and established a model by introducing a correction factor that contained the charge’s moving velocity (*V*), azimuth angle (*θ*) and scaled distance (*Z*) to Baker’s formula. However, only the overpressure was studied, while the arrival time and impulse were not considered. At the same time, due to the meshing problem, we can observe that the pressure curve is too smooth and the peak pressure is reduced. Meir Mayseless and Eli Bar-on [23] conducted a numerical study to analyze the effect of a moving charge on a moving target. They focused on the reflected pressures and the overall force on the target. In general, the numerical investigations mentioned above were based on a bare spherical TNT moving charge explosion, where the approaches were overly idealistic and unsupported by experimental data.

Most investigations on shock waves focused on stationary charge explosions, while the studies on moving charge explosion shock wave pressure were mainly based on numerical simulations. Only a small number of reports were published in the open literature that describe the experimental research of a spherical moving charge explosion. The reports were mainly focused on the spherical charge explosion under ideal circumstances. In this study, we focused on the experimental investigation of a cylindrical charge explosion, which is closer to a real moving charge explosion situation. The objective of the study was to measure and analyze the shock wave of moving charge and stationary charge explosions accurately. The comparative analysis was adopted to study the differences and evaluate the power of a moving charge explosion. The correlation model method was proposed and verified using experimental data and was used to study the overpressure relationship between the stationary charge and moving charge explosions. It provided a theoretical analysis basis for the shock wave pressure of moving charge and stationary charge explosions.

This paper is organized as follows: in Section 2, we describe the arrangement of the whole experiment setup and the design of the pressure measurement system; in Section 3, the results of the experimental analysis and comparison with calculation results are given, and the difference between the stationary charge explosion and moving charge explosion is discussed. The conclusions and perspectives of this work are given in Section 4.

## 2. Experiment

The aim of the experimental setup was to measure the shock wave pressure parameters of a moving charge explosion and compare the results with a stationary charge explosion. A smoothbore cannon was used to load the charge horizontally in order to control the charge so that it burst at a given speed in a specific location. The different firing velocities were controlled by changing the propellant mass, and the loading time was adjusted according to the specific test. According to the relationship of propellant mass, initial velocity and terminal velocity of the moving charge, the location of the blast point was predetermined. To reasonably arrange the pressure sensors to measure a moving charge explosion, it was necessary to carry out stationary charge tests to obtain the blast wave pressure distribution. Combined with the stationary charge test data and the predicted blast point, the blast wave measuring points of a moving charge explosion could be set up. The experiment conditions were as follows: (a) the ambient temperature was 25 °C and (b) no wind.

### 2.1. Experimental Materials and Set-Up

In the experiments, cylindrical charges with the same structure were used in stationary charge and moving charge explosions, and a DHL explosive (which consisted of 80% desensitized RDX with 20% aluminum powder) was pressed inside the charges. The material of the charge casing was steel. The specific parameters of the charge are shown in Table 1.

**Figure 1:**
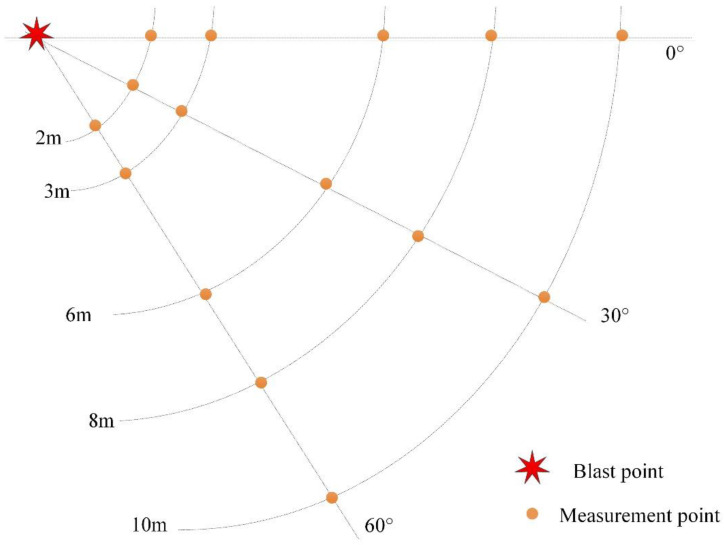
Schematic of the measurement points in the stationary charge explosion experiment.

In the static explosion experiment, a detonator and booster were used to initiate the charge. The charge was placed horizontally on a wooden base 2 m above the ground. The axis of the charge was required to coincide with the theoretical ballistic line of a moving charge explosion. Measurement points were arranged at 2 m, 3 m, 6 m, 8 m and 10 m distances from the blast point in 0°, 30° and 60° directions, as shown in Figure 1.

**Figure 2:**
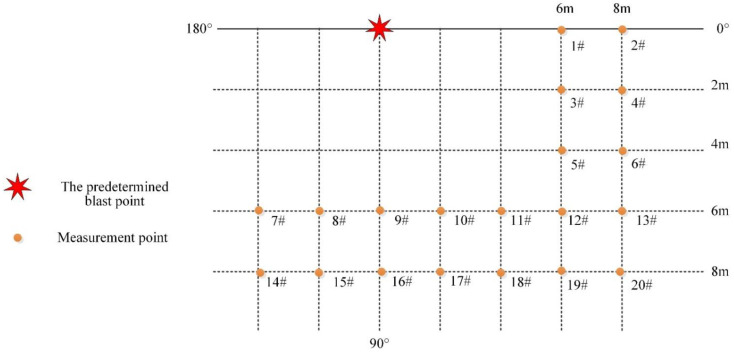
Schematic of the measurement points in the moving charge explosion experiment.

In the moving charge experiment, a smoothbore cannon was used to horizontally launch the charge at a certain velocity. The height of the fire line was 2 m, and the angle between the charge and the horizontal plane was 0°. Different from the stationary charge test, due to the uncertainty of the moving charge blast point, the array layout of the measurement points was more effective at obtaining the blast wave pressure. The predetermined blast center ground projection point was set as the coordinate origin. The positive direction of the charge movement was the 0° direction, and the reverse direction was the 180° direction. The nearest measurement point along the center was 6 m and the distance between two adjacent sensors of the same measurement line was 2 m, as shown in Figure 2.

The location of the blast center in the moving charge explosion needed to be determined again after the test. The layout of the measurement points in the stationary charge explosion experiment was not suitable for the subsequent comparative analysis. Therefore, we needed to conduct the stationary explosion test again using the moving charge explosion test site to facilitate the analysis of the pressure signal at the same distance measurement points.

### 2.2. Testing System

**Figure 3:**
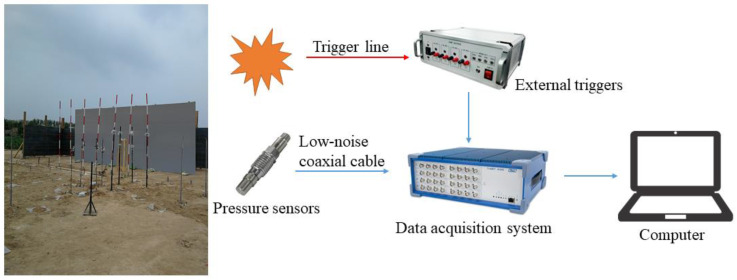
Schematics of the test system and a photo of the experiment site.

The shock wave pressure test system consisted of the following parts: shock wave pressure sensors, a data acquisition system, an external trigger and a low-noise coaxial cable. The shock wave pressure sensor was connected with the data acquisition system through a low-noise coaxial cable. The signals could be immediately conditioned, acquired and stored using the data acquisition system within the ICP signal conditioner. An external trigger was used to provide the zero-time signal of the explosion. In the experiment, one end of the trigger line was wound on the charge, and the other end was connected to the input of the external trigger. The output end of the external trigger was connected to the signal input end of the data acquisition system through a coaxial cable. The external trigger signal and pressure signal were collected at the same time, and the arrival time of the external trigger signal was taken as time zero. The experiment results were saved on a computer. Table 2 lists the devices and their parameters used in the experiments. The schematics of the test system and the photo of the experiment site are shown in Figure 3.

### 2.3. Experimental Results

**Figure 4:**
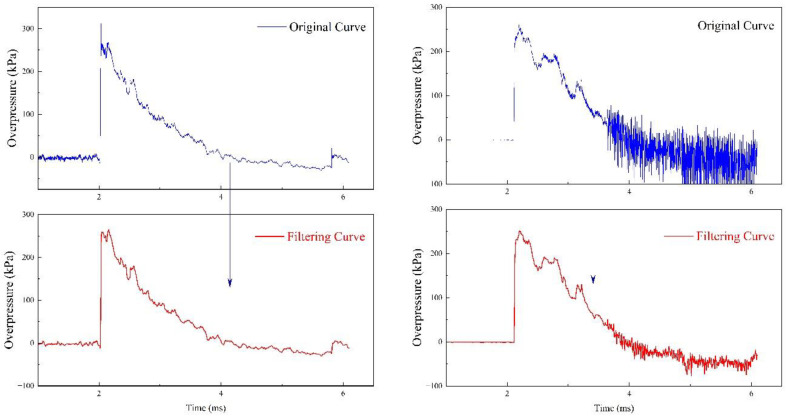
Original curves and filtered curves of the shock wave pressure.

The blast wave pressure signal usually contained trend terms, singularities and high-frequency noise; therefore, the signal was moderately filtered and smoothed in this study. In this study, we adopted the wavelet method to analyze the signal energy spectrum. The upper limit frequency of the signal was less than 40 kHz. After analysis of the signal, we used a Butterworth low-pass filter with an upper cut-off frequency of 40 kHz to eliminate noise [24]. Figure 4 shows the original and filtered overpressure time history curves in the stationary charge explosion experiment.

**Figure 5:**
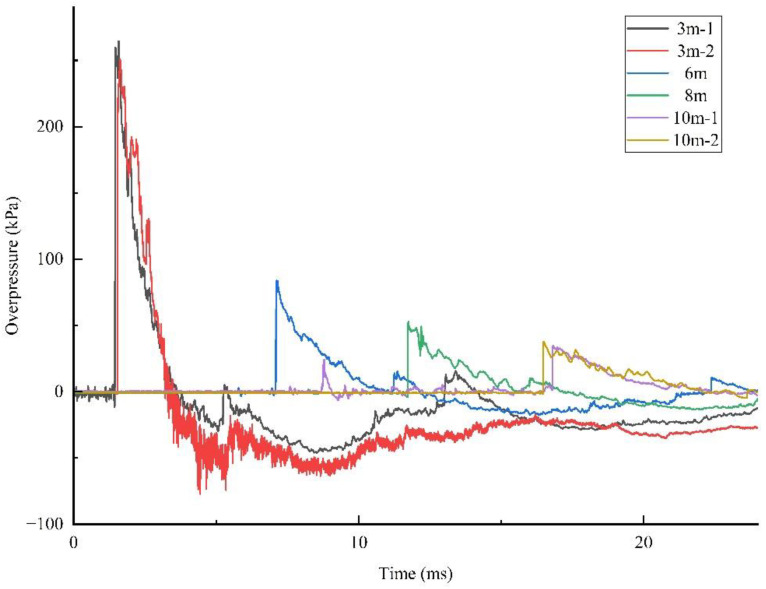
Shock wave overpressure curves of a stationary charge explosion.

The typical blast wave pressure curves at different measurement points are shown in Figure 5. Some curves are not drawn here because they contained significant noise that interfered with the viewing of other curves. The shock wave parameters of one stationary charge explosion test are listed in Table 3. Other experimental data are listed in Table A1 in Appendix A. It can be seen that the values of the overpressure, arrival time and impulse were all similar for the same distance from different directions. The shock wave pressure distribution was uniform in the stationary experiment. The charge explosion produced a high number of fragments, and it was not easy to obtain shock wave impulse and duration data.

**Figure 6:**
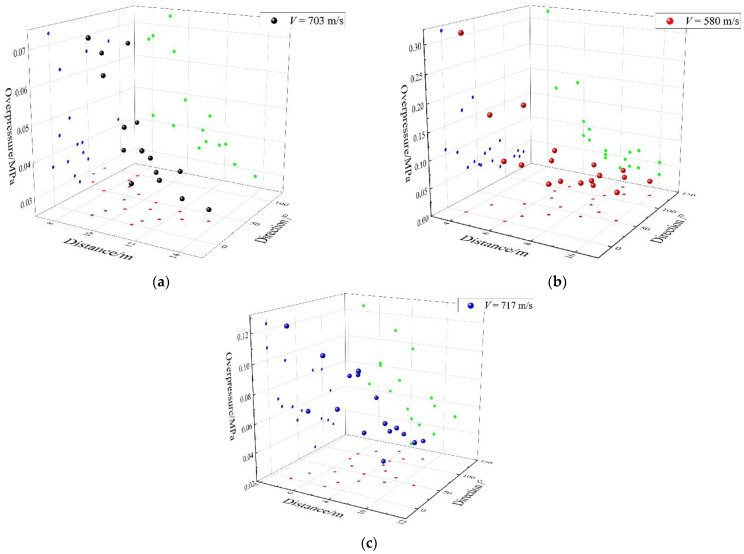
Distribution of the shock wave overpressure from moving charge explosions: (**a**) the speed was 703 m/s and the deviation was −3 m (in the opposite direction of the charge motion), (**b**) the speed was 580 m/s and the deviation was +2 m (in the positive direction of the charge motion) and (**c**) the speed was 717 m/s and the deviation was +1 m.

Moving charge explosion experiments were carried out with the same charge in the stationary charge experiment. A high-speed camera system was used to obtain the terminal velocity of the charge and the deviation of the actual blast point from the predicted blast center. The details of the high-speed camera were as follows: (1) resolution: 1280 × 800, (2) distance from the blast center: 150 m and (3) framerate: 5000 fps. Three experiments at different velocities were carried out, where the terminal velocity of the charge and the deviation were as follows: (1) the speed was 703 m/s and the deviation was −3 m (in the opposite direction of the charge’s motion), (2) the speed was 580 m/s and the deviation was +2 m (in the positive direction of the charge’s motion) and (3) the speed was 717 m/s and the deviation was +1 m. The shock wave overpressure distributions of different moving charge experiments are graphed in Figure 6. The experiment data of the moving charge explosions are presented in tabular form in Appendix A. Table A2, Table A3 and Table A4 include shock wave pressure parameters of the different moving charge tests. It can be seen that the shock wave overpressure of the moving charge explosion was influenced by distance and direction at the same time. In general, when the distance from the blast center increased, the overpressure value decreased. However, the shock wave overpressure values had a large difference at the same distance from different directions. The detailed analysis is discussed in Section 3.

## 3. Analysis

### 3.1. Analysis of the Shock Wave Pressure in the Stationary Charge Explosion Experiment

In order to calculate the shock wave pressure parameters using empirical formulas, the weight of the confined cylindrical charge was converted into the weight of the bare charge according to the relevant formula. After the calculation, the equivalent bare charge weight was substituted into the empirical formulas to calculate the shock wave pressure at different distances [25]. In this study, Henrych’s empirical formula was used to calculate the shock wave pressure at different distances [9]. It is generally believed that in the case of an infinite air explosion, the following formula is satisfied:

(1) $$\frac{h}{\sqrt[{3}]{\omega_{e}}}\geq 0.35$$

where *h* is the height of the charge from the ground (m) and $\omega_{e}$ is the TNT equivalent (kg); furthermore:

(2) $$\Delta p=\frac{1.379}{\bar{r}}+\frac{0.543}{{\bar{r}}^{2}}-\frac{0.035}{{\bar{r}}^{3}}+\frac{0.0006}{{\bar{r}}^{4}}\begin{array}{cc} & (0.05\leq \end{array}\bar{r}\leq 0.3)\;\Delta p=\frac{0.607}{\bar{r}}-\frac{0.032}{{\bar{r}}^{2}}+\frac{0.209}{{\bar{r}}^{3}}\begin{array}{cc} & (0.3\leq \end{array}\bar{r}\leq 1)\;\Delta p=\frac{0.065}{\bar{r}}+\frac{0.397}{{\bar{r}}^{2}}+\frac{0.322}{{\bar{r}}^{3}}\begin{array}{cc} & (1\leq \end{array}\bar{r}\leq 10),$$

where Δp is the peak value of overpressure (MPa) and $\bar{r}=r/\sqrt[{3}]{\omega_{e}}$ is the scaled distance (m/kg^1/3^), where *r* is the distance of the gauge point from the blast center (m).

The experimental shock wave overpressure value was taken as the average value of different directions. Table 4 lists the calculated values $X_{i}$ and the measured values of two tests.

**Figure 7:**
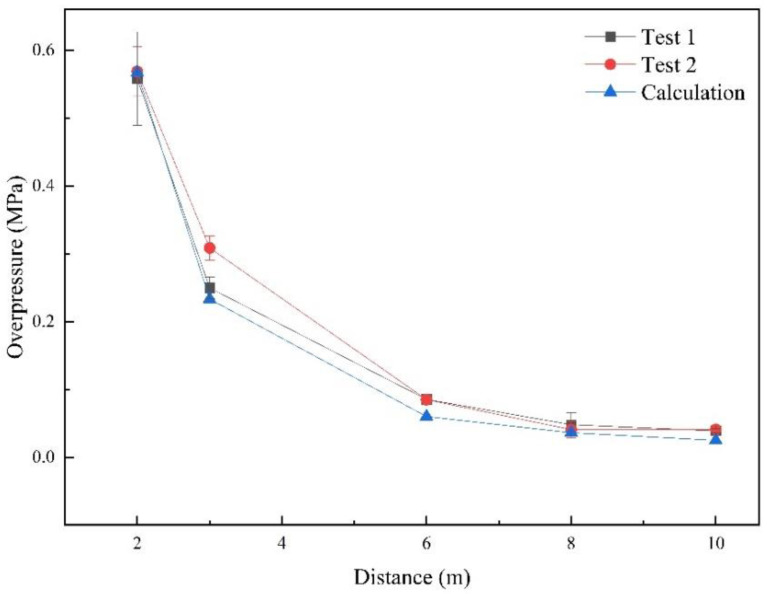
The relationship between overpressure and distance.

The overpressure values of two stationary charge explosion experiments were graphed and analyzed, and the attenuation curves compared with the calculation are shown in Figure 7. In the two stationary charge explosion experiments, the average value of the overpressure values in different directions was used as the overpressure at this distance. The error bar was calculated using the deviation of the overpressure in three directions (0°, 30°, 60°) shown in Table 3. It can be seen that the pressure distribution of the two tests presented a relatively uniform law and the test data were in good agreement with the theoretical calculation. The theoretical calculation values were smaller than the test data, mainly because the equivalent used in the theoretical calculation was the TNT equivalent that was converted using the charge under the ideal condition. In general, Henrych’s empirical formula could describe the stationary charge blast wave pressure field within a certain range in this research.

### 3.2. Analysis of the Shock Wave Pressure in the Moving Charge Explosion Experiment

**Figure 8:**
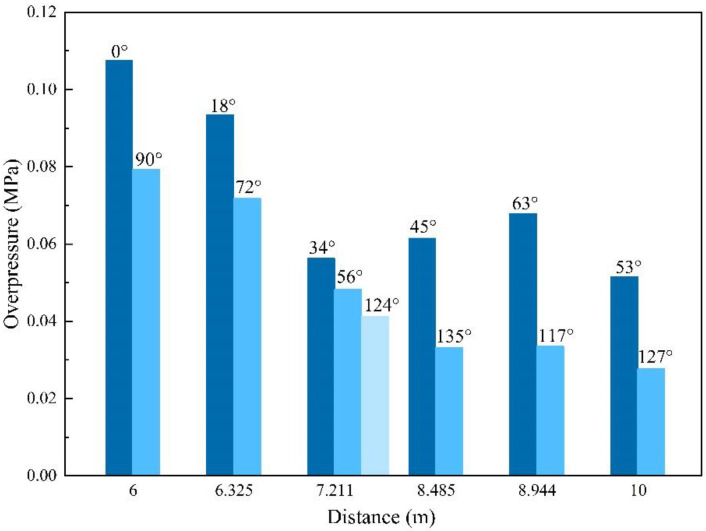
Overpressure values of different directions in the moving charge explosion with a charge speed of 580 m/s.

**Figure 9:**
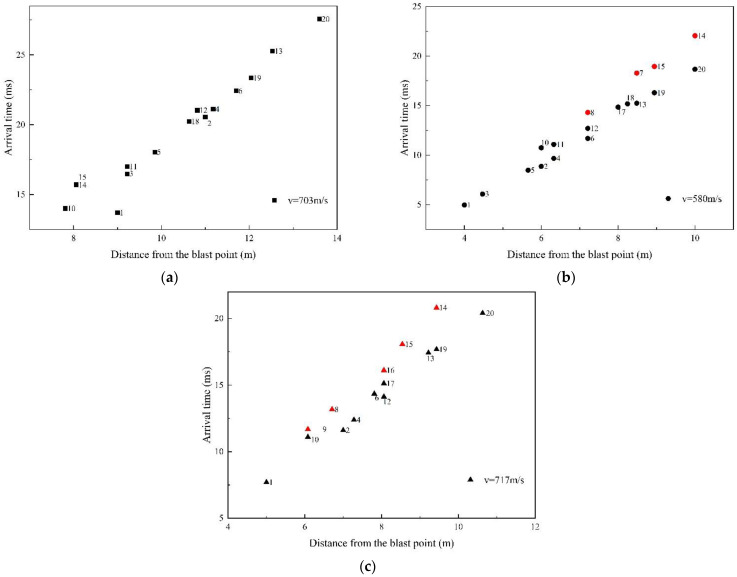
Arrival time at different measurement points from a moving charge explosion: (**a**) the speed was 703 m/s and the deviation was −3 m (in the opposite direction of the charge motion), (**b**) the speed was 580 m/s and the deviation was +2 m (in the positive direction of the charge motion) and (**c**) the speed was 717 m/s and the deviation was +1 m. The measurement points with a direction angle greater than 90° are marked in red.

The shock wave pressure parameters of the moving charge explosions are listed in Table A2, Table A3 and Table A4 in Appendix A. From Figure 6, it can be seen that the shock wave overpressure of a moving charge explosion was influenced by the distance and direction at the same time. For example, in the moving charge experiment with a charge speed of 703 m/s, the overpressure values of measurement points #3 and #11 were 0.0625 MPa and 0.0396 MPa, respectively, and the gain was 57.83%; the overpressure values of measurement points #14 and #15 were 0.0418 MPa and 0.0665 MPa, respectively, and the gain was 59.09%. Differences also existed in the impulse and the duration. In the moving charge experiment of 580 m/s, there were multiple sets of measurement points at the same distance in different directions, as shown in Figure 8. It can be seen as the direction angle increased, the value of overpressure decreased; for example, the overpressure value of point #2 in the 0° direction was 35.69% higher than that of point #10 in the 90° direction at 6 m. Figure 9 shows the arrival time at different measurement points from a moving charge explosion. In general, the arrival time increased with the distance from the blast center. At those measuring points with a direction angle greater than 90° (marked in red in Figure 9), the arrival time was greater. This indicated that the movement of the charge affected the propagation of the shock wave. The results showed that the shock wave characteristics of different directions were influenced differently by the movement of the charge.

In this study, the theory of velocity vector superposition was adopted to study the shock wave characteristics. The velocity of the center of the blast wave could be predicted by applying the principle of conservation of linear momentum. Thus, the momentum of the explosive prior to detonation was set equal to the momentum of the explosion gases and air contained by the spherical blast wave. Neglecting the mass of air displaced by the explosive charge, the law of conservation of linear momentum is as follows:

(3) $$m_{e}\nu_{0}=\left(m_{e}+m_{a}\right)\nu_{c}=\left(m_{e}+\frac{4}{3}\pi \rho_{a}r^{3}\right)\nu_{c}$$

where $m_{e}$ is the weight of the explosive, $v_{0}$ is the terminal velocity of the charge, $m_{a}$ is the weight of air contained within the spherical blast wave, $v_{c}$ is the velocity of the center of the blast wave, $\rho_{a}$ is the density of air and *r* is the radius of the spherical blast wave.

Since $m_{e}=4\pi \rho_{e}a^{3}/3$, the velocity of the center of the shock wave is given as follows:

(4) $$\nu_{c}=\frac{m_{e}\nu_{0}}{m_{e}+\frac{4}{3}\pi \rho_{a}d^{3}}=\frac{\nu_{0}}{1+\frac{\rho_{a}}{\rho_{e}}r^{3}}$$

where $\rho_{e}$ is the density of charge and *a* is the radius of the charge.

**Figure 10:**
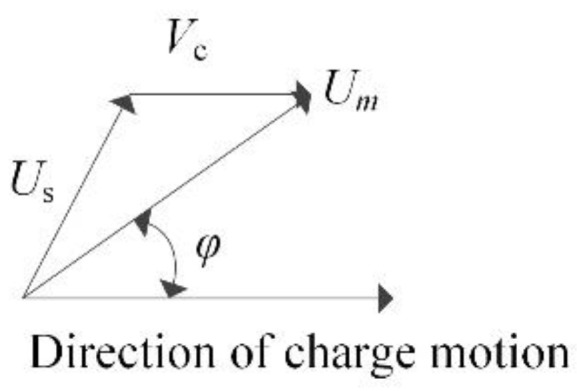
Model of velocity vector superposition.

The distance $r_{c}$ that the center has traveled (in the direction of original charge motion) from the point of detonation at any time *t* is given by Equation (5). The velocity of the surface of the spherical blast wave produced by the moving charge is shown in Figure 10 and Equation (6).

(5) $$rc=\int_{0}^{t}v_{c}dt$$

(6) $$\vec{U_{m}}=\vec{v_{c}}+\vec{U_{s}}$$

where $\vec{U_{m}}$ is the shock wave velocity at distance *r* and angle *φ*, $\vec{U_{s}}$ is the shock wave velocity for a stationary charge and *φ* is the angle from the line of flight for a moving charge (referred to as the center of the shock wave).

**Figure 11:**
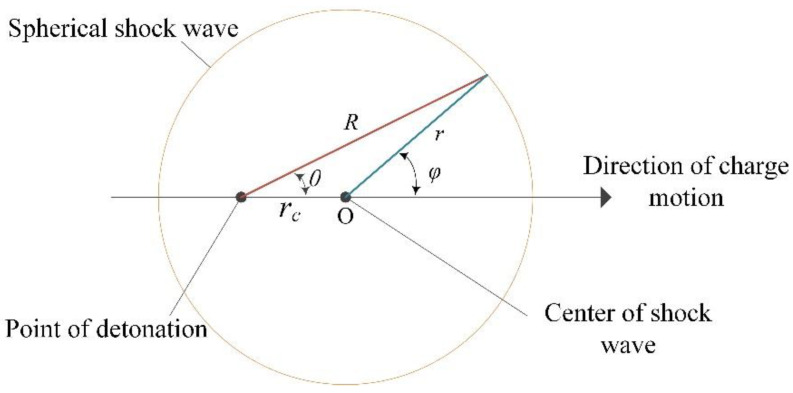
Shock wave model of a moving charge explosion.

According to the assumptions of the moving charge explosion shock wave field, the shock wave model was established in Figure 11. The distance and angle from the center were as follows:

(7) R=rccosθ+(r2−rc2sin2θ)12, sinφ=Rrsinθ

According to the equation of Rankine–Hugoniot [26], there is a relationship between the shock wave velocity and shock wave overpressure, as given in Equation (8). The shock wave pressure of a moving charge explosion can be calculated by considering a stationary charge explosion and the shock wave velocity given by Equation (9):

(8) $$\frac{P}{P_{0}}=\frac{2k}{k+1}\left(\frac{U^{2}}{c_{0}{}^{2}}-1\right)$$

(9) $$\frac{P_{m}}{Ps}=\frac{U_{m}{}^{2}-c_{0}{}^{2}}{U_{s}{}^{2}-c_{0}{}^{2}}$$

where P and $P_{0}$ are the shock wave pressure and atmospheric pressure, respectively; k is the specific heat capacity of air; U is the shock wave velocity; $c_{0}$ is the air sound velocity of the shock wave front; $P_{m}$ and $P_{s}$ are the shock wave pressures of moving charge and stationary charge explosions, respectively; and $U_{m}$ and $U_{s}$ are the shock wave velocities of moving charge and stationary charge explosions, respectively.

### 3.3. Comparative Analysis of the Moving Charge and Stationary Charge Explosions

**Figure 12:**
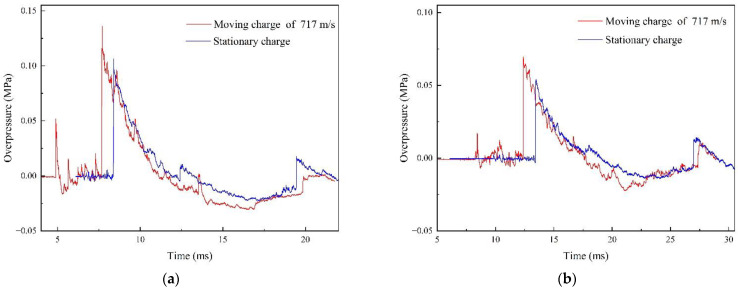
Shock wave overpressure–time history curves of the moving charge and stationary charge explosions: (**a**) test point #1 at 5 m in the 0° direction and (**b**) test point #4 at 7.28 m in the 16° direction.

In order to analyze the difference in shock wave characteristics between moving charge and stationary charge explosions, the same experimental site and measurement points used in the moving charge experiment were used to carry out another stationary charge test. In order to ensure the same measurement distances in the two different experiments, we adopted a high-speed camera to determine the deviation of the actual blast point from the predicted blast center in the moving charge explosion experiment. Then, the stationary charge was detonated at the same blast center and measurement points were arranged at the same locations as in the moving charge explosion experiment. The blast center was set at the explosion center of the moving charge when the speed was 717 m/s and the deviation was +1 m. Figure 12 shows the shock wave pressure curves of moving charge and stationary charge explosions at the same measurement points.

From the pressure–time history curves, we can see the following: (1) There existed secondary pressure peaks in the shock wave pressure at measurement points # 1 and # 4; and the main reason for this was that the afterburning reaction of aluminum powder released a certain amount of energy [27]. (2) The basic shape of the moving charge explosion shock wave curve was consistent with the stationary charge; however, before the arrival of the moving charge explosion shock wave signal, there was an obvious disturbance signal in the waveform. After the explosive expansion, the head of the ammunition was stung, the body expanded and deforms, cracks occurred and the windward area increased. At this time, an oblique shock wave or even a positive shock wave with a stronger air compression effect was generated at the ammunition head. The crack in the body also produced a strong disturbance to the supersonic incoming flow and generated a shock wave.

From the analysis of the shock wave pressure in Section 3.2, we can find that there was a relationship between the moving charge and stationary charge explosions. The small amount of experimental data was not enough to construct a valid engineering model. Instead, we adopted a correlation model between the moving charge and stationary charge explosions based on numerical simulation data referring to the investigation of Haiyan Jiang [28]:

(10) $$P_{m}={\left(1+\frac{0.3}{1+\bar{R}}\frac{v_{0}}{c_{0}}\cos\theta \right)}^{2}P_{s}$$

where $P_{m}$ is the shock wave overpressure of moving charge explosion, $\bar{R}$ is the scaled distance, $v_{0}$ is the velocity of moving charge, $c_{0}$ is the air sound velocity of the shock wave front, and θ is the angle between measurement line direction and charge moving direction.

**Figure 13:**
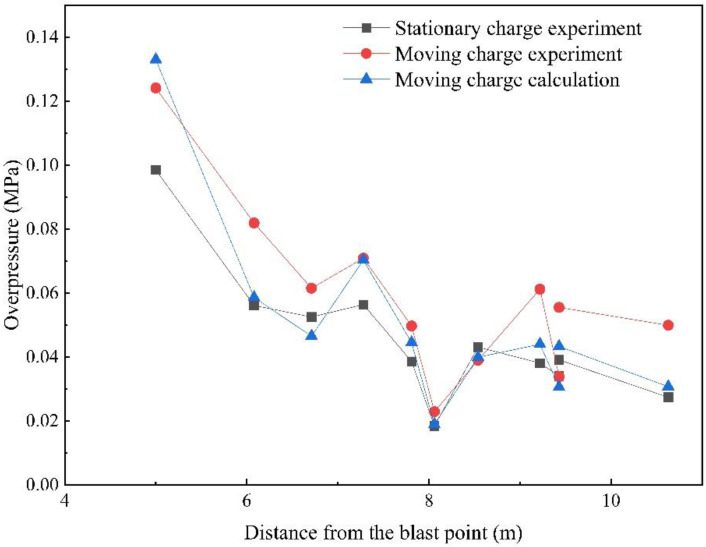
Overpressure values at different distances from the blast center for the stationary charge experiment, moving charge experiment and moving charge calculations.

The shock wave overpressure values of the stationary charge experiment, moving charge experiment, moving charge calculation and the errors are listed in Table A5 in Appendix A. The overpressure values with the distance from the blast center are graphed in Figure 13. From the data in Table A5 and Figure 13, we can find that the peak overpressure of a moving charge at the same measurement point was greater than that of the stationary charge in the direction between 0° and 90°, and the values increased by 25%–60%. However, at measurement points #14 and #15 where the directions were 112° and 111°, the overpressure values were smaller than those of the stationary charge. This indicated that the influence of the moving charge explosion was different in different directions. It can be seen that the measured overpressure data and the model calculation results were in good agreement in addition to individual measurement points. The measured values and theoretical values were close, and the average error was 16.53%. The relationship was based on the simulation data of the TNT spherical bare charge under ideal conditions. When the model was applied to the experimental analysis, the type of explosives and the existence of the shell affected the calculation results, though the error was within acceptable limits. Therefore, the correlation model based on the velocity vector superposition method could describe the overpressure relationship between the stationary charge and moving charge explosions.

## 4. Conclusions

In this study, the shock wave pressures of explosions were studied using an experimental method. The experimental set-up and measurement system was designed for moving charge explosions and could ensure that the moving charge exploded at a certain velocity in the predetermined location. The shock wave pressure curves and parameters (overpressure, arrival time, duration, impulse) could be obtained accurately at different distances in different directions.

The shock wave pressure distribution of the moving charge explosions had a strong directionality, and the pressure parameters were influenced by the charge’s moving velocity, direction angle and distance from the blast center. The overpressure value of a moving charge was greater than that of a stationary charge at angles between 0° and 90°, where the values were 25–60% higher.

For the stationary charge explosion, Henrych’s empirical formula was used to calculate the shock wave pressure at different distances, which could describe the stationary charge shock wave pressure field. For the moving charge explosion, the correlation model based on a velocity vector superposition method could describe the overpressure relationship between the stationary charge and moving charge explosions, and the average error was 16.53%. The experimental data showed that the charge’s moving velocity had a large influence on the shock wave pressure parameters.

## Figures and Tables

**Table 1:** Parameters of the charge.

Explosive Type	Mass of the Explosive (kg)	Power/TNT Equivalent	Mass of the Charge (kg)	Charge–Weight Ratio
DHL	3.4	148%	22.5	0.15

**Table 2:** Parameters of the devices used in the experiments.

Device	Characteristic Parameters	
**Pressure sensors**	Manufacturer and type	PCB 113B
	Measurement range	50–500 psi
	Resonant frequency	≥500 kHz
	Non-linearity	≤1.0% FS
**Data acquisition system**	Manufacturer and type	Elsys TraNET FE
	Maximum sampling rate	20 MHz
**External triggers**	Resolution	16 bits
	Manufacturer and type	Self-developed
	Synchronization time error	<0.1 μs

**Table 3:** Shock wave pressure parameters of a stationary charge explosion.

*r* (m)		2	3	6	8	10
**Arrival time (ms)**	0°	1.9205	3.1502	-	13.6763	18.1702
	30°	1.879	3.2418	8.7969	13.4221	18.4423
	60°	1.7312	3.1431	-	13.6873	18.4981
**Overpressure (MPa)**	0°	0.4893	0.2713	-	-	0.0396
	30°	0.5331	0.2337	0.0852	0.0568	0.0419
	60°	0.6520	0.2438	-	0.0640	0.0347
**Duration (ms)**	0°	-	1.9869	-	-	5.5762
	30°	-	1.678	3.836	3.761	5.9199
	60°	1.0326	-	-	-	5.8608
**Impulse (Pa·s)**	0°	-	207.3613	-	-	86.0357
	30°	-	227.7904	112.5334	80.6452	74.1739
	60°	273.9726	-	-	-	72.8959

**Table 4:** Comparison of the static explosion overpressure between the test and calculation results.

r		2	3	6	8	10
Δp **(MPa)**	Test 1	0.5581	0.2496	0.0852	0.0480	0.0387
	Test 2	0.5687	0.3085	0.0849	0.0498	0.0410
	$X_{i}$	0.5670	0.2332	0.0599	0.0361	0.0249

## Appendix A

**Table A1:** Shock wave parameters of two stationary charge explosion experiments.

No.	Distance (m)	Direction (°)	Overpressure (MPa)		Arrival Time (ms)		Duration (ms)		Impulse (Pa·s)	
			Test 1	Test 2	Test 1	Test 2	Test 1	Test 2	Test 1	Test 2
1	2	0	0.4893	0.6054	1.9205	2.0638	-	-	-	-
2	2	30	0.5331	0.5320	1.879	2.1975	-	-	-	-
3	2	60	0.6520	-	1.7312	-	1.0326	-	273.9726	-
4	3	0	0.2713	0.2941	3.1502	3.4705	1.9869	2.2384	207.3613	214.4894
5	3	30	0.2337	0.2976	3.2418	3.5745	1.678	2.0873	227.7904	224.1726
6	3	60	0.2438	0.3338	3.1431	3.3208	-	2.0572	-	153.2446
7	6	0	-	0.0882	-	9.3054	-	4.867	-	121.0561
8	6	30	0.0852	0.0816	8.7969	9.1315	3.836	3.8277	112.5334	108.3134
9	6	60	-	-	-	-	-	-	-	-
10	8	0	-	-	13.6763	-	-	-	-	-
11	8	30	0.0568	0.0513	13.4221	13.5703	3.761	5.6484	80.6452	89.5161
12	8	60	0.0640	0.0484	13.6873	13.7765	-	-	-	-
13	10	0	0.0396	0.0355	18.1702	18.7394	5.5762	5.709	86.0357	88.8154
14	10	30	0.0419	0.0411	18.4423	18.6595	5.9199	6.1173	74.1739	69.9258
15	10	60	0.0347	0.0465	18.4981	18.6195	5.8608	5.1924	72.8959	68.7210

**Table A2:** Shock wave parameters of a moving charge explosion experiment (*V* = 703 m/s, *x* = −3 m).

No.	Distance (m)	Direction (°)	Overpressure (MPa)	Arrival Time (ms)	Duration (ms)	Impulse (Pa·s)
1	9.000	0	0.0729	13.714	4.0526	85.9353
2	11.000	0	0.0363	20.5518	5.5646	84.2359
3	9.220	13	0.0625	16.475	5.3599	103.7210
4	11.180	10	0.0444	21.1205	5.2788	81.6215
5	9.849	24	0.0482	18.044	5.614	101.6906
6	11.705	20	0.0359	22.4256	5.739	80.6452
7	6.083	99	-	-	-	-
8	6.083	81	-	-	-	-
9	6.708	63	-	-	-	-
10	7.810	50	0.0654	14.0149	4.574	90.7206
11	9.220	41	0.0396	17.0041	4.9231	80.3966
12	10.817	34	0.0397	21.036	5.1138	68.5965
13	12.530	29	0.0308	25.266	5.7071	67.7507
14	8.062	97	0.0418	15.6894	4.9037	66.0066
15	8.062	83	0.0665	15.718	-	-
16	8.544	69	-	-	-	-
17	9.434	58	-	-	-	-
18	10.630	49	0.0339	20.2386	4.905	72.7995
19	12.042	42	0.0365	23.3482	6.187	60.6878
20	13.601	36	0.0283	27.5653	-	-

**Table A3:** Shock wave parameters of a moving charge explosion experiment (*V* = 580 m/s, *x* = +2 m).

No.	Distance (m)	Direction (°)	Overpressure (MPa)	Arrival Time (ms)	Duration (ms)	Impulse (Pa·s)
1	4.000	0	0.3166	4.973	3.5723	229.1607
2	6.000	0	0.1076	8.8689	3.4358	-
3	4.472	27	0.167	6.0749	3.4358	-
4	6.325	18	0.0935	9.675	4.5174	136.0359
5	5.657	45	0.1834	8.4901	-	-
6	7.211	34	0.0563	11.696	4.6671	114.2473
7	8.485	135	0.0332	18.289	5.7971	66.7557
8	7.211	124	0.0413	14.3129	5.0428	84.8176
9	6.325	108	-	-	-	-
10	6.000	90	0.0793	10.7511	3.6718	90.7206
11	6.325	72	0.0718	11.0868	-	-
12	7.211	56	0.0483	12.7069	4.4979	82.3158
13	8.485	45	0.0615	15.2499	4.9943	88.0759
14	10.000	127	0.0277	22.0549	5.6498	52.8053
15	8.944	117	0.0335	18.9459	4.3389	56.2667
16	8.246	104	-	-	-	-
17	8.000	90	0.0266	14.8509	3.692	26.9796
18	8.246	76	0.0457	15.1879	-	-
19	8.944	63	0.0679	16.3119	5.0801	74.174
20	10.000	53	0.0515	18.6739	5.9499	72.896

**Table A4:** Shock wave parameters of a moving charge explosion experiment (*V* = 717 m/s, *x* = +1 m).

No.	Distance (m)	Direction (°)	Overpressure (MPa)	Arrival Time (ms)	Duration (ms)	Impulse (Pa·s)
1	5.000	0	0.1241	7.6924	3.4291	171.8705
2	7.000	0	0.108	11.6038	-	-
3	5.390	22	0.0645	-	-	-
4	7.280	16	0.0709	12.3864	5.175	122.4323
5	6.400	39	-	-	-	-
6	8.060	30	-	14.1269	-	-
7	7.810	130	-	-	-	-
8	6.710	117	0.0615	13.1714	3.6121	56.5451
9	6.080	99	0.0801	11.6785	-	-
10	6.080	81	0.0819	11.0933	-	-
11	6.710	63	-	-	-	-
12	7.810	50	0.0497	14.3434	5.2028	96.0351
13	9.220	41	0.0612	17.4136	3.8273	81.3008
14	9.430	122	0.0338	20.8034	4.659	52.8053
15	8.540	111	0.039	18.0594	4.1001	56.2667
16	8.060	97	0.0426	16.1012	4.3131	69.512
17	8.060	83	0.0229	15.1299	3.941	26.9796
18	8.540	69	-	-	-	-
19	9.430	58	0.0555	17.6737	5.4401	74.174
20	10.630	49	0.0499	20.3965	-	-

**Table A5:** Experimental and calculated shock wave overpressures.

No.	Distance (m)	Direction (°)	Overpressure of Stationary Charge Experiment (MPa)	Overpressure of Moving Charge Experiment (MPa)	Overpressure of Moving Charge Calculation (MPa)	Error (%)
1	5.000	0	0.0985	0.1241	0.1330	7.20
4	7.280	16	0.0564	0.0709	0.0703	−0.84
8	6.710	117	0.0525	0.0615	0.0465	−24.36
10	6.080	81	0.0561	0.0819	0.0586	−28.47
12	7.810	50	0.0386	0.0497	0.0445	−10.49
13	9.220	41	0.0381	0.0612	0.0440	−28.02
14	9.430	122	0.0340	0.0338	0.0306	−9.57
15	8.540	111	0.0431	0.039	0.0399	2.25
17	8.060	83	0.0184	0.0229	0.0189	−17.45
19	9.430	58	0.0391	0.0555	0.0433	−22.06
20	10.630	49	0.0274	0.0499	0.0307	−31.14
